# Rapid Screening of the Epidermal Growth Factor Receptor Phosphosignaling Pathway via Microplate-Based Dot Blot Assays

**DOI:** 10.1155/2012/473843

**Published:** 2012-08-15

**Authors:** Amedeo Cappione, Janet Smith, Masaharu Mabuchi, Timothy Nadler

**Affiliations:** EMD Millipore, Merck KGaA, 17 Cherry Hill Drive, Danvers, MA 01923, USA

## Abstract

Expression profiling on a large scale, as is the case in drug discovery, is often accomplished through use of sophisticated solid-phase protein microarrays or multiplex bead technologies. While offering both high-throughput and high-content analysis, these platforms are often too cost prohibitive or technically challenging for many research settings. Capitalizing on the favorable attributes of the standard ELISA and slot blotting techniques, we developed a modified dot blot assay that provides a simple cost-effective alternative for semiquantitative expression analysis of multiple proteins across multiple samples. Similar in protocol to an ELISA, but based in a membrane bound 96-well microplate, the assay takes advantage of vacuum filtration to expedite the tedious process of washing in between binding steps. We report on the optimization of the assay and demonstrate its use in profiling temporal changes in phosphorylation events in the well-characterized EGF-induced signaling cascade of A431 cells.

## 1. Introduction

**Figure 1:**
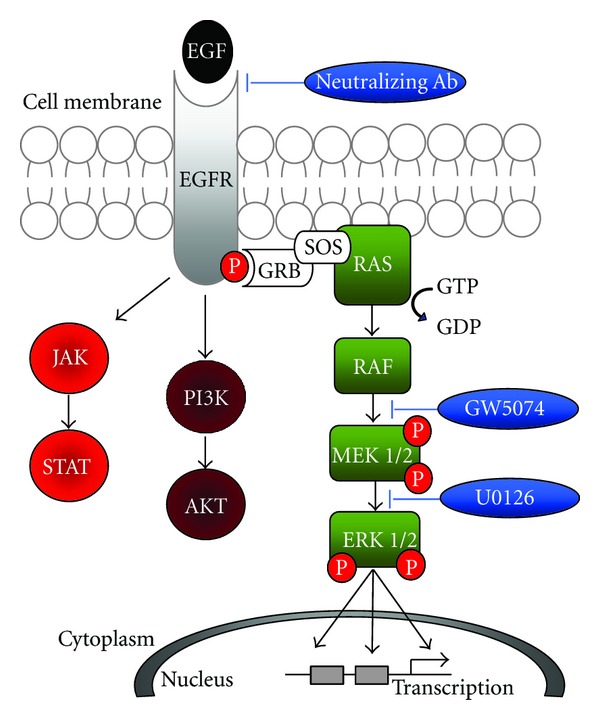
The EGFR signaling cascade. The binding of EGF to the EGF receptor (EGFR) results in receptor dimerization and conformational changes triggering autophosphorylation. Under proper conditions, phosphorylated EGFR activates any number of three downstream signaling pathways through Ras, PI3K, and JAK, respectively. The work performed in this study focuses on the Ras cascade (highlighted in green). The three inhibitors (blue) are shown acting at their specific sites of signal interruption.

Signaling through receptor tyrosine kinases (RTKs) is a highly conserved cellular mechanism, controlling fate determination, proliferation, survival, and migration [1, 2]. In most instances, ligand binding initiates conformational changes in the externally facing receptor molecule leading to autophosphorylation on the internal portion of the receptor. A subsequent chain of phosphorylation events propagates the signal to the nucleus culminating in the transcription of genes required to direct changes in cell function (the EGFR cascade is outlined in Figure 1). Given the dynamic interplay of cells with their surrounding microenvironment and owing to the presence of a myriad of other simultaneously activated paths, this process must be tightly regulated to ensure proper responses occur. The broad importance of RTK signaling is highlighted by the well-documented role of pathway dysregulation in human disease, most notably cancer. RTK mutations have been implicated in a variety of cancers, specifically, members of the epidermal growth factor receptor (EGFR) family in brain, lung, and breast cancer. In fact, thirty percent of all solid tumors possess Ras or Raf mutations, including almost 90% of pancreatic adenocarcinomas [3, 4]. 

Due to the inherent complexity of the global signaling network and the involvement of their constituents in malignancy, these pathways have been extensively studied by researchers looking for insight into the mechanisms underlying both normal and aberrant growth. Detecting alterations in phosphorylation patterns within signaling profiles is a technique commonly employed to map the dose dependence and specificity of small-molecule inhibitors targeting upstream components. Traditionally, compound screening was performed in cell-free assays using purified enzymes as the target. More recently, a greater significance has been placed on the use of cell-based approaches where multiple components in a single pathway and multiple signaling cascades can be monitored simultaneously. Such analyses require complex and expensive detection platforms such as flow cytometers or high-content imaging systems. While well suited for the high throughput needs of large-scale screens at the industrial level, such platforms may not fit the workflow or demand of smaller research groups. Alternative detection systems offering semiquantitative measurement of multiple proteins in parallel include the enzyme-linked immunosorbent assay (ELISA), multiplex bead arrays, western blots, and slot blots. Although plate based, a fact that simplifies setup and signal detection, ELISAs require a pair of protein-specific antibodies which identify unique epitopes and are also quite time consuming due to multiple binding steps and extensive washing. Slot blotting apparatuses have been developed that offer increased throughput over standard western blots yet retain the same overall labor-intensive protocol that is not amenable to automation. In addition, signal quantitation for both blotting techniques is limited by the dynamic range of the developing film and method of densitometric analysis.

In this paper, we present a modified dot blotting technique for protein detection where purified proteins or cell lysates are applied directly to membrane-based 96-well microplates. This dot blot assay combines the plate-based ease of handling offered by ELISAs with vacuum filtration to greatly expedite the process of semiquantitative analysis of protein expression. Following the addition of protein sample, a two-step antibody binding process using an HRP-conjugated secondary detection antibody is performed. In the final step, the conversion of chemiluminescent substrate provides the signal in each well that can be quantified using a standard plate reader. For a 96-well plate, the entire process, from sample addition to data acquisition, requires less than 90 minutes. Our assay was validated using lysates derived from the extensively studied EGFR signaling cascade of the A431 epidermal carcinoma cell line. Temporal changes in phosphoactivation of three proteins (EGFR, MEK1/2, and ERK1/2) were measured at five-minute intervals across a twenty-minute time course of EGF stimulation. Pathway mapping was further interrogated using a set of three site-specific inhibitors of the EGFR cascade.

## 2. Materials and Methods

### 2.1. Cell Culture

 A431 (CRL-1555, ATCC, Manassas, VA), a human skin carcinoma cell line, was maintained in complete media (DMEM + 10% FBS) and passaged routinely by trypsinization (TrypLESelect, GIBCO/Life Technologies, Grand Island, NY) to ensure log phase growth. For induction experiments, 100 K live cells were seeded per well in 6-well plates, cultured for 2 days, and then serum-starved for 20 HR. Following synchronization, cells were exposed to 100 ng/mL human EGF (Cell Signaling Technology, Danvers, MA) for 5–20 minutes. For inhibitor studies, cultures were pre-incubated with 10 *μ*m U0126 (Cell Signaling Technology), 5 *μ*m GW5074 (Sigma-Aldrich, St. Louis, MO), or 10 *μ*g/mL anti-EGFR neutralizing Ab (EMD Millipore), for 2 hrs prior to EGF exposure. Following induction, total cell lysates were prepared as described below (for the EGFR pathway schematic, see Figure 1).

### 2.2. Cell Counting and Viability

 10 *μ*L sample was mixed with 190 *μ*L guava ViaCount reagent and incubated for 5 minutes at RT. Sample data was acquired on a guava easyCyte HT instrument and analyzed using guava ViaCount software (all EMD Millipore).

### 2.3. Cell Lysis

 Lysis was performed using two buffers: (1) Cytobuster Protein Extraction Reagent (EMD Millipore) and a modified RIPA buffer (25 mM Tris-HCl pH 7.6, 150 mM NaCl, 1% NP-40, 1% sodium deoxycholate, and 0.1% SDS). All buffers were supplemented with protease inhibitors and phosphatase inhibitors (EMD Millipore). All buffers were chilled on ice prior to use. Following induction, cells were washed twice with ice-cold PBS. 400 *μ*L of lysis buffer was added to each well. Samples were incubated on ice for 5 minutes with occasional swirling. To pellet cellular debris, resulting extracts were centrifuged at 16000 g × 15 min at 4°C. Cell lysates were removed, aliquoted, and stored at −20°C until assayed.

### 2.4. IR-Based Protein Quantitation

 Proteins were quantified using the Direct Detect assay-free sample card and Direct Detect Spectrometer (EMD Millipore). Each card contained four hydrophilic polytetrafluoroethylene (PTFE) membrane positions, each surrounded by a hydrophobic ring to retain analyzed sample within the device's IR beam. All measurements were performed using 2 *μ*L of sample per membrane position. A “buffer only” sample was also analyzed as a reference blank. Sample concentration was determined in reference to a calibration method. For all experiments, the system was initially calibrated using National-Institute-of-Standards-&-Technology- (NIST-) certified BSA SRM927d in phosphate-buffered saline (PBS). A series of ten concentration points (0.125–5 mg/mL) was used to generate the instrument calibration curve.

### 2.5. Antibody Validation

One of the limiting factors in biochemistry is the availability and quality of antibodies. Prior to use in the dot blot assay, each candidate antibody was subjected to a stringent validation procedure [5]. As part of the initial screening process, we reviewed the certificate of analysis for each of the four antibodies: anti-phospho-EGFR (TYR1069, clone 9H2), anti-phospho-Mek1/2 (SER218/SER222, clone E237), anti-phospho-Erk 1/2 (THR202/TYR204, clone 12D4), and anti-GAPDH (clone 6C5) employed in this study (all Abs are from EMD Millipore). All four were validated for use in western blotting analysis as part of the standard quality control testing by EMD Millipore. Staining with each of the four antibodies resulted in detection of a single prominent band at the approximate molecular weight and the lack of any nonspecific binding. In addition, all four were validated using lysates derived from A431 cells, the only cell line employed in this study. Antibodies against phosphorylated epitopes had to demonstrate specificity to stimulated (ex. EGF) or inhibited (anti-EGF neutralizing Ab) to yield phosphorylated (signal) or nonphosphorylated forms (no signal) of the protein, respectively.

### 2.6. Dot Blot Protocol (see Figure 2)

Prewet PVDF membrane Multiscreen plates (EMD Millipore) with 100 uL 70% Ethanol for 15 seconds then immediately wash 2X with 100 *μ*L Milli-Q H_2_0 by vacuum filtration using the Multiscreen_HTS_ Vacuum Manifold (EMD Millipore) with pressure set to 4′′Hg. In all wash steps use vacuum filtration. Add diluted lysate (50 *μ*L/well) and incubate for 30 minutes at RT on a plate shaker at low speed. Wash plates 2X with Tris-Buffered Saline (TBS). Block sample wells in 0.5% nonfat dried milk (in TBS) for 5 minutes on the shaker then remove blocking agent by vacuum filtration. Add 50 *μ*L/well diluted primary antibody and incubate on a shaker for 10 minutes. Each antibody was previously titrated to optimize performance. The antibodies included anti-phospho-EGFR (TYR1069, clone 9H2), anti-phospho-Mek1/2 (SER218/SER222, clone E237), anti-phospho-Erk1/2 (THR202/TYR204, clone 12D4), and anti-GAPDH (clone 6C5). Wash plates 3X with TBS + 0.1% Tween-20 (TBST). Add 50 *μ*L/well diluted goat anti-rabbit IgG HRP (EMD Millipore) and incubate on a shaker for 10 minutes. Wash plates 3X as above. Add 100 *μ*L/well of Luminata Forte Western HRP Reagent (EMD Millipore) and incubate for 5 minutes on a shaker. Read signal using BioTek Synergy microplate reader (BioTek, Winooski, VT). For each well, chemiluminescent signal was measured and presented as counts per second (CPS).

## 3. Results

**Figure 3:**
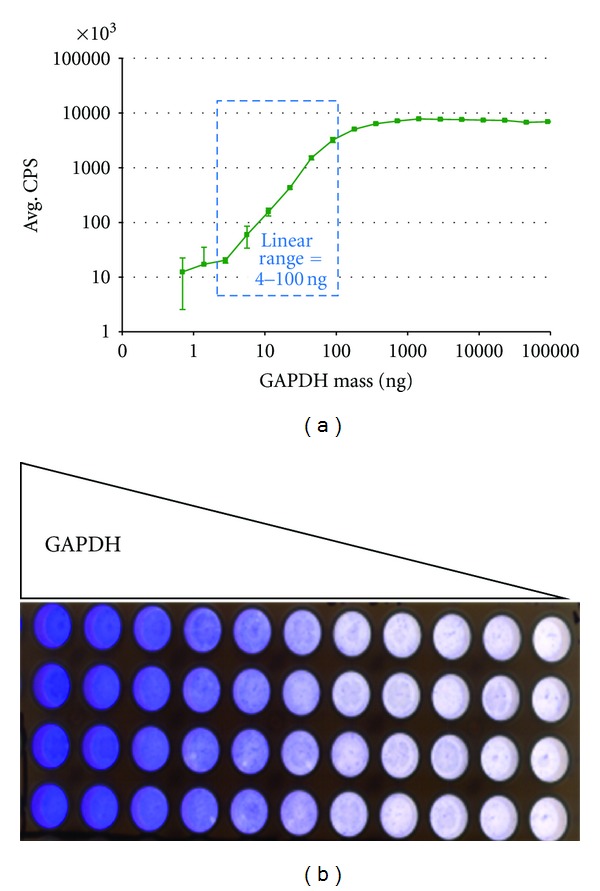
Dot blot assay feasibility—titration of pure GAPDH. (a) The titration curve demonstrates a range 4–100 ng input protein whereby linear signal could be detected. Each point represents the mean of 4 replicates. (b) Coomassie staining of membrane wells after protein loading. Four replicates are shown for each concentration point.

Initial feasibility studies and optimization of the dot blot assay were performed using purified Glyceraldehyde-3-Phosphate Dehydrogenase (GAPDH) protein in PBS buffer. Representative results from a serial dilution of GAPDH are presented in Figure 3. From the graph, the assay was able to detect down to ~4 ng and was linear in response up to 100 ng protein loaded. The assay shows a robust signal:noise ratio and does not appear to be limited by binding capacity of the membrane; the theoretical binding capacity of a single well (0.3 cm^2^) of membrane is approximately 90 *μ*g [6]. The assay was optimized as follows for each step: 30 min protein binding, 10 min primary Ab binding, and 10 min secondary Ab binding. All binding steps were performed with low-speed agitation at room temperature. Overall assay time is 90 minutes. It is possible that protein range could be increased with greater Ab input or increased binding reaction times although this may lead to elevations in nonspecific binding. Due to differences in binding kinetics, optimization of reaction conditions may be required for each protein analyzed.

For the dot blot assay to have broader application, it must perform in the context of total cell lysates where relative protein concentration, buffering conditions, and lysate clarity may impact not only membrane binding but also antibody detection characteristics. To assess such issues, we first extracted lysates from A431 cells using two distinct lysis buffers, a modified RIPA buffer and a nondetergent-based commercial extraction reagent. Following extraction, protein samples were quantified using the Direct Detect IR-based quantitation system. On average, the RIPA buffer liberated five times greater total protein than the nondetergent buffer (data not shown); this may be due to the presence of harsher detergent in the RIPA buffer resulting in greater protein solubilization.

**Figure 4:**
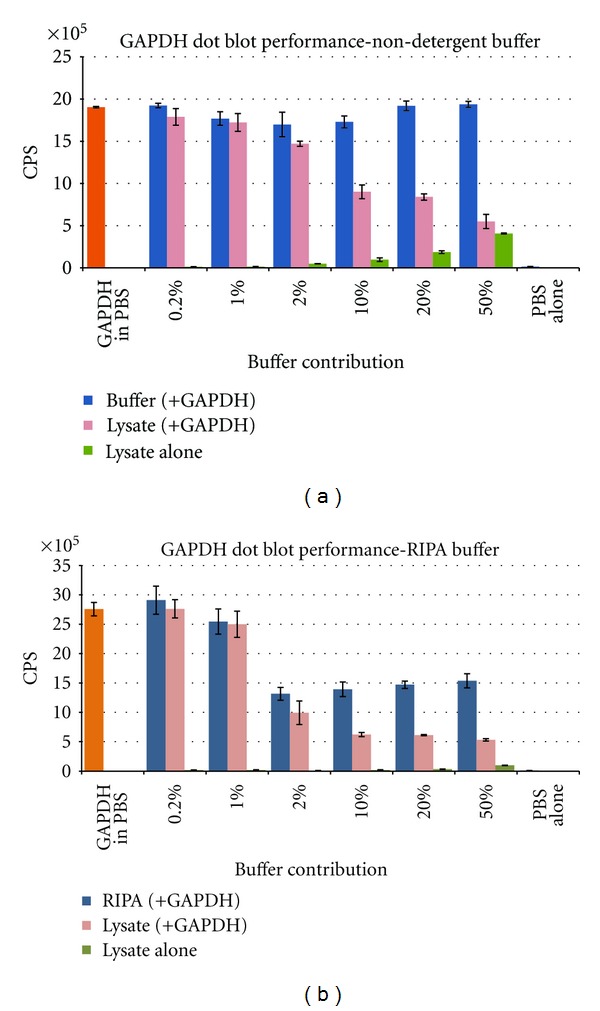
Impact of lysis buffer components on dot blot performance—Detergents. Lysates (pink bars) or buffers alone (blue) were diluted in PBS, spiked with 100 ng GAPDH and assayed for changes in GAPDH detection. The green bars show detection of the native GAPDH present in A431 lysates (no GAPDH was spiked into these samples). The CPS signal for GAPDH in PBS is displayed by the orange Bar. Each bar represents the average of 3 individual replicates. Protein concentrations for the lysates used were determined to be (a) nondetergent buffer, 494 ng/*μ*L, and (b) RIPA buffer, 2443 ng/*μ*L. The RIPA buffer was diluted to 494 ng/*μ*L prior to setup. Total protein lysate loaded was as follows: 0.2% = 49 ng; 1% = 295 ng; 2% = 494 ng; 10% = 2950; 20% = 4940 ng; 50% = 12350 ng.

 Total lysates from each extraction condition were used to assess the potential effects of protein concentration and buffer components on dot blot assay performance. The results of this experiment are outlined in Figure 4. Briefly, lysates, or buffers alone, were diluted to varying degrees with PBS. Samples were spiked with 100 ng purified GAPDH and loaded onto microplates. A standard dot blot assay was then performed. For the RIPA buffer, any contribution greater than 1% (0.5 *μ*L in 50 *μ*L reaction volume) caused a significant decrease in GAPDH signal; this is most likely due to detergents interfering with membrane binding and limiting protein-protein interactions. By contrast, the nondetergent-based buffer alone had little or no effect on GAPDH signal even at 50% sample dilution; this result may be important for situations where either total protein concentrations are low or the protein of interest is expressed at relatively low levels. We also found a reduction in GAPDH signal in both buffer types when lysate load/well was increased. Signal reduction was slightly greater in the homebrew samples due to the contributing detergent effect. Signal loss is most likely due to protein crowding and/or competition for membrane binding. More importantly, native GAPDH was easily detected in nondetergent-derived samples with signal >100X over background for 12.5 *μ*g lysate loaded. 

**Figure 5:**
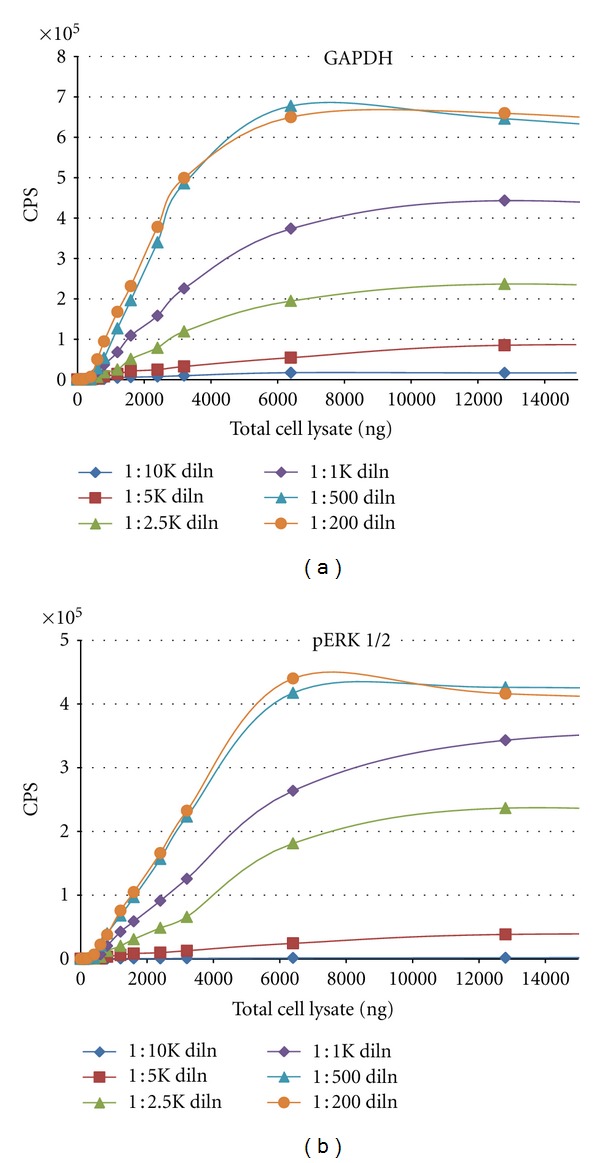
Native protein detection—primary antibody optimization. Serum-starved A431cells were stimulated with EGF for 20 min and harvested and lysates prepared as described previously. Dot blot assays were performed to optimize detection by (a) GAPDH and (b) phospho-ERK1/2 antibodies. A series of seven lysate concentrations (100–10000 ng/well) was used to assess six different dilutions of each primary antibody. All points were run in triplicate.

We sought to determine the assay's linear range and define the optimal concentration of primary antibody required for protein detection. Given the wide variability in relative protein expression within a cell and between different cells, such optimization may be required for each protein (and each cell type's lysate) to be measured. The graphs in Figure 5 depict the results for titration curves performed on EGF-stimulated A431 lysates using antibodies specific for the house-keeping GAPDH protein and phosphorylated ERK1/2. The dot blot assay demonstrated a linear range of detection for 400–6000 ng/well. Irrespective of primary Ab concentration, little to no signal could be detected below 400 ng lysate. The assay also failed to detect any greater signal levels in wells with ≥6000 ng lysate. In fact, at higher sample loads, counts per second (CPS) values tended to decrease; this was most likely due to the higher complexity of the lysate solution as well as increased competition for membrane binding. In both cases, a 1 : 500 dilution of primary antibody (2 *μ*g/mL GAPDH Ab, 1 *μ*g/mL pERK1/2 Ab) provided optimal detection.

**Figure 6:**
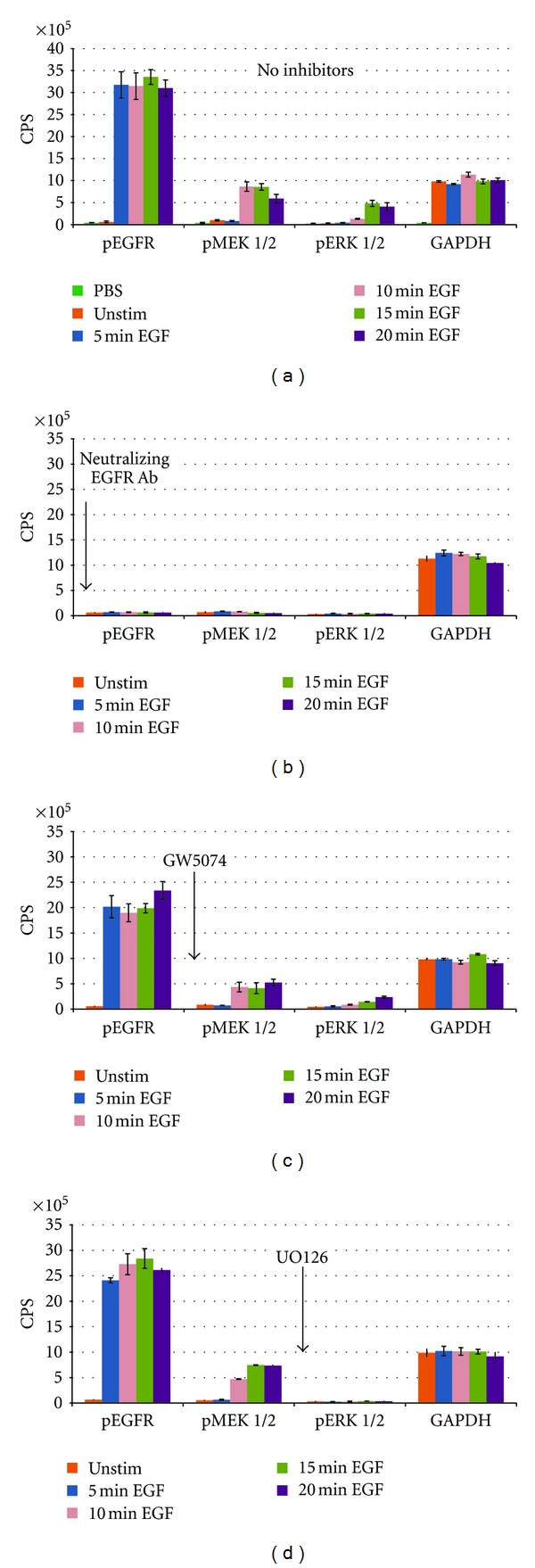
Temporal mapping of the EGFR cascade using site-specific inhibitor molecules. The four bar graphs represent results for the following: (a) EGF stimulation, no inhibitor, and EGF stimulation in the presence of (b) neutralizing EGFR Ab, (c) GW5074—an inhibitor of Raf kinase activity, and (d) U0126—a highly selective inhibitor of MEK1/2. For each inhibitor, the arrow indicates the point of pathway inhibition. Each graph contains the time course (0–20 minutes) of expression profiles for each of the four proteins analyzed. Each bar value represents the average of three replicates.

We next applied the dot blot assay in a proof-of-concept study to track changes in protein phosphorylation for the EGFR signaling cascade of A431 cells cultured with EGF. Ligand binding activates a chain of signaling events, which includes successive phosphorylation of the EGFR, MEK, and ERK proteins. Given the temporal nature of phosphoactivation within the cascade, synchronized A431 cells were exposed to EGF and harvested at 5 minute intervals for a total of 20 minutes. The time course was performed with EGF stimulus alone and in the presence of three pathway inhibitors. In the absence of inhibition, a clear temporal order of phosphorylation events is seen (Figure 6). EGF stimulation resulted in an almost immediate increase in the presence of phosphorylated EGFR, which was maintained at high levels for the entire 20 minutes. Among the four proteins measured, the phospho-EGFR signal was by far the highest level detected; this finding is not unexpected given that the A431 cell line expresses abnormally high levels of EGFR [7]. Phosphorylated Mek1/2 was first detected at 10 minutes followed by ERK1/2 at 15 minutes. For the latter two proteins, the phosphorylated state was far more transient appearing to decrease soon after initial appearance. GAPDH detection was included in each data set as a loading control to permit cross-sample comparisons. Overall, for 60 GAPDH wells analyzed across 20 experimental conditions (3 replicates for each), the mean CPS value was 1,026,671 ± 87,405 with a coefficient of variation of 8.5%. Pre-incubation with the EGFR blocking antibody completely abolished all downstream phosphorylation events. U0126 treatment prevented phosphorylation of ERK1/2 without affecting either upstream event. At the concentration applied, GW5074 caused only partial inhibition of MEK1/2 and ERK1/2. Interestingly, GW5074 also caused a reduction in EGFR phosphorylation suggesting a potential positive feedback loop involving intermediary signaling proteins. A more extensive study involving challenges with various concentrations of GW5074 may offer greater insight into this phenomenon.

## 4. Discussion

Standard dot blotting is a method of protein detection similar to the western blot technique but differing in that protein samples are not initially separated electrophoretically on polyacrylamide gels but simply applied directly onto the membrane's surface. Once applied, proteins are driven to bind the membrane through either active pressure (vacuum) or gentle agitation and passive absorption. Dot and slot blotting techniques have been used extensively by molecular biology researchers to affix proteins and nucleic acids on membranes for the purpose of quantitation, DNA homology assessment, protein-DNA/RNA interactions, enzymatic activity, and the study of ligand-receptor binding. The standard device is comprised of three main parts: the upper block with an array of slots for sample loading, a middle component that holds the inserted membrane, and a bottom block with a connector permitting vacuum filtration. A set of screws clamps the assembled device in place thereby minimizing sample bleed-over. Given this format, slot blots offer greater throughput capacity than the standard western blot and are therefore ideal for screening applications. However, the device's main value is in sample loading; all subsequent steps are performed in the same labor-intensive manner as a western blot. To expedite the process without sacrificing throughput, our modified dot blot takes advantage of 96-well microplates equipped with PVDF membrane. The plate-based format minimizes sample bleed-over and permits easy reagent loading at each step. Since the plate is membrane based, all wash steps can be performed via vacuum filtration. The plate format and simple reaction steps are also well suited for automation and expanded screening needs. A final benefit is being able to use a standard plate reader for chemiluminescent signal detection; this format offers greater dynamic range than film densitometry enhancing quantitative capacity of the assay.

The work presented here clearly demonstrates the feasibility of the plate-based dot blot application for semiquantitative detection or comparative analyses of multiple proteins and/or multiple samples in parallel. The assay performed well on pure protein samples but more importantly worked for total cell lysates although the linear ranges of detection were considerably different. The assay is, however, quite sensitive to detergent interference, an important consideration when choosing extraction reagents. As well, samples with high viscosity or large amounts of debris had a tendency to cause a reduction in filtration flow rate and, in more severe cases, complete clogging of the membrane. Dilution of viscous samples or precentrifugation to clear particulates ameliorated clogging issues. In summary, the dot blot assay offers a cost-effective protein expression screening tool for researchers with moderate throughput needs.

## Figures and Tables

**Figure 2:**
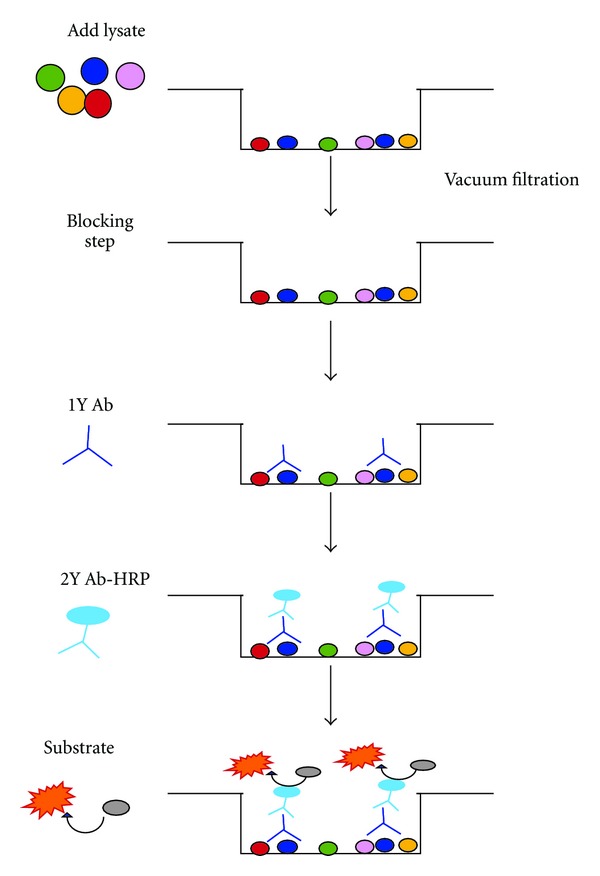
The dot blot protocol. Depicted in the diagram is the assay for one representative well from a 96-well microplate. After protein binding, a blocking step is performed to reduce nonspecific Ab binding to any unoccupied region of membrane. The remaining portion of the assay is similar to ELISA except that all wash steps are performed via vacuum filtration.
